# Effect of Wetting Agents on Microhardness, Color, and Gloss of a Nanohybrid Composite after Aging

**DOI:** 10.1590/0103-644020266842

**Published:** 2026-08-07

**Authors:** Valeria Bisinoto Gotti, Rafael Martins Chagas, Isabela Torres Resende, João Vitor da Silva Duarte, Marcella Bessa Palhares, Analia Gabriella Borges Ferraz Facury, César Penazzo Lepri, Vinícius Geraldo Martins, Ana Paula Ayres Oliveira

**Affiliations:** 1 Restorative Dentistry, University of Uberaba Dental School, Uberaba, MG, Brazil

**Keywords:** composite resins, dental sealants, dental materials

## Abstract

This study investigated whether wetting agent application alters surface microhardness, gloss, and color stability of a nanohybrid resin composite after simulated aging by brushing or thermocycling. Sixty discs of one nanohybrid resin composite were fabricated and divided into three conditions: no wetting agent, Wetting Resin (WR), or Modeling Resin (MR). Baseline evaluations included Knoop microhardness, color using the CIE Lab* system, and 60° gloss. Aging was performed by either standardized brushing or 10,000 thermocycles (5-55 °C). Post-aging values were compared with baseline data. Statistical analysis used two-way repeated-measures ANOVA/Tukey for microhardness, and nonparametric tests for gloss and ΔE (α = 0.05). Wetting agent application lowered baseline microhardness compared to controls, with MR showing the lowest values. Thermocycling reduced gloss, while brushing increased gloss in WR and maintained it in MR. Sealants also affected color stability, with material- and method-dependent responses. In conclusion, the wetting agent influenced the mechanical and optical behavior of the nanohybrid resin composite in a manner dependent on the agent and aging protocol. These materials reduced surface hardness and produced distinct gloss and color responses to brushing versus thermocycling.

## Resumo

Este estudo investigou se a aplicação de resinas umidificadoras altera a microdureza da superfície, o brilho e a estabilidade de cor de um compósito nanohíbrido após envelhecimento simulado por escovação ou termociclagem. Sessenta discos de um compósito nanohíbrido foram fabricados e divididos em três condições: sem resina modeladora, Wetting Resin (WR) ou Modeling Resin (MR). As avaliações iniciais incluíram microdureza Knoop, cor usando o sistema CIE Lab* e brilho a 60°. O envelhecimento foi realizado por escovação padronizada ou 10.000 termociclos (5-55 °C). Os valores após o envelhecimento foram comparados com os dados iniciais. A análise estatística foi realizada por ANOVA dois fatores com medidas repetidas/Tukey para microdureza e testes não paramétricos para brilho e ΔE (α = 0,05). A aplicação de resinas umidificadoras reduziu a microdureza inicial em comparação ao grupo controle, com o grupo MR apresentando os menores valores. A termociclagem reduziu o brilho, enquanto a escovação aumentou o brilho no grupo WR e o manteve no grupo MR. As resinas modeladoras também afetaram a estabilidade da cor, com respostas dependentes do material e do método. Concluindo, resinas umidificadoras influenciaram o comportamento mecânico e óptico do compósito nanohíbrido de maneira dependente do agente e do protocolo de envelhecimento. A aplicação destes materiais reduziu a dureza da superfície e produziu respostas distintas de brilho e cor à escovação em comparação à termociclagem.

**Figure:**
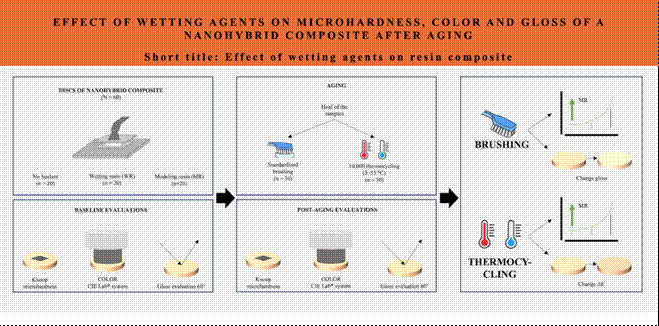

## Introduction

Recent advances in esthetic adhesive dentistry have led to restorative materials that closely mimic the look of natural tooth structures. A major clinical challenge remains the restoration of anterior teeth, where clinicians must re-create shape, color, and function while also reproducing key optical features of enamel and dentin ^1^, such as fluorescence ^2^ ^,^ ^3^, opalescence ^4^ ^,^ ^5^, translucency ^6^, and surface gloss ^7^, among other properties. The objective is for the restorative resin composite to resemble healthy tooth structures as much as possible, while simultaneously providing reliable adhesion and mechanical characteristics compatible with the tissues it is intended to replace ^8^ ^,^ ^9^.

In direct restorations, low-viscosity modeling liquids are often applied to reduce resin composite sticking to instruments or brushes and to facilitate a smoother surface finish ^10^ ^,^ ^11^. These materials share the same resin matrix as resin composites but contain fewer filler particles. Besides facilitating sculpting and adaptation to cavity walls, they may function as surface sealants, filling superficial microporosities and thereby enhancing stain resistance, wear resistance, and marginal sealing ^12^.

Evidence in the literature is still inconsistent regarding the influence of modeling liquids on the long-term optical and mechanical behavior of resin composite restorations. Some investigations indicate that the reduced filler content may lower surface microhardness ^13^, whereas others have shown benefits such as improved marginal adaptation and enhanced resistance to staining under simulated conditions like thermocycling or brushing ^10^ ^,^ ^14^. These divergent outcomes highlight the importance of additional studies, especially considering the growing clinical application of these agents.

In this context, the present study aimed to evaluate the influence of two modeling liquids on the optical properties and microhardness of a nanohybrid resin composite. Furthermore, the performance of these compositions following simulated toothbrushing and thermocycling regimens was investigated. The null hypotheses tested were that wetting agents' application, mechanical brushing, and thermocycling do not affect: [1] the surface microhardness; [2] the color stability; and [3] the gloss of a nanohybrid resin composite.

## Material and methods

### Material and specimen preparation

This in vitro study followed the CRIS reporting guidelines ^15^. A 3×2 factorial design was adopted, considering two independent variables: wetting agent and aging method. The nanohybrid resin composite (Forma, shade A1E, Ultradent Products, UT, USA) and the wetting agents (WR: Wetting Resin, Ultradent Products, UT, USA; MR: Modeling Resin, BISCO, IL, USA) are detailed in Table 1. Sample size (N = 60) was determined based on pilot data and calculated with G*Power software version 3.1.7 (Franz Faul, Kiel University, Germany), adopting a significance level of α = 0.05 and statistical power of 0.9. 

**Table 1:** Resin composite and wetting agents tested.

Material (lot number)	Manufacturer	Composition
Forma (D0EOQ)	Ultradent Products, South Jordan, UT, USA	Matrix: TEGDMA, UDMA, chromium iron oxide, Lumilux blue; Filler: silicon dioxide, zirconium dioxide
Wetting Resin (WR; D0F8D)	Ultradent Products, South Jordan, UT, USA	TEGDMA^1^, Bis-GMA^2^
Modeling Resin (MR; 2100003422)	Bisco, Inc., Schaumburg, IL, USA	UDMA^3^, Bis-EMA^4^, TEGDMA^1^, Bis-GMA^2^, amorphous silica

Composition and indications of the materials are from the manufacturer’s information.

^1^TEGDMA: Triethylene Glycol Dimethacrylate; ^2^Bis-GMA: Bisphenol A Dimethacrylate; ^3^UDMA: Urethane Dimethacrylate; ^4^Bis-EMA: Ethoxylated Bisphenol A Glycol Dimethacrylate.

Sixty (n = 10) disc-shaped specimens of shade A1E (6 mm in diameter × 4 mm in height) were produced by a single trained operator, with care taken to minimize bubble incorporation. For fabrication, a metallic mold was filled with a single uncured Forma resin composite increment and covered with a polyester Mylar strip (TDV, SC, Brazil). In 40 randomly selected specimens, one drop (0.05 mL per drop) ^16^ ^,^ ^17^ of wetting agents (WR or MR) was applied to the uncured resin composite using a calibrated micropipette (LABMATE Soft; HTL Lab Solutions, Warsaw, Poland). The material was then applied homogeneously to the entire surface of the resin composite using a spatula (Safident, Cosmedent, IL, USA) for 5 s and covered again with a Mylar strip. The remaining 20 discs (controls) received no sealant. To achieve uniform thickness and remove excess resin composite, a glass plate of 1 mm thickness was lightly pressed on top of the Mylar using fingertip pressure. All specimens were light-cured according to the manufacturers’ instructions for 20 s through both the glass and polyester strips using an LED curing unit (Valo, Ultradent Products Inc., UT, USA) in standard mode (≈ 990 ± 5 mW/cm²; 20 J/cm²). Light output was verified after every five samples with a radiometer (Demetron, Kerr Dental, CA, USA). After polymerization, the samples were demolded and immersed in distilled water at 37 °C for 24 h. The polishing procedure was not performed, and any sample presenting surface voids or irregularities was discarded and replaced with a flat, bubble-free specimen. 

**Figure 1:**
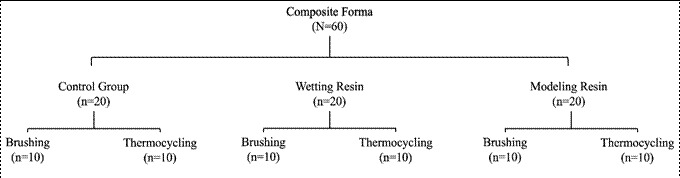
Distribution of the specimens (n = 10) according to the wetting agent and aging method.

The specimens were fabricated and allocated as shown in Figure 1. Half of the samples from each group (n =10) were subjected to the aging challenge (brushing or thermocycling), and all the samples were evaluated before and after the aging challenge. 

### Knoop Microhardness evaluation

Surface microhardness was measured on the top of each specimen using a tester (HMV-2, Shimadzu Corporation, Tokyo, Japan) equipped with a Knoop diamond indenter. Five indentations, evenly distributed across the surface, were produced using a 100-g load applied for 5 seconds. The mean value of these measurements was taken as the Knoop hardness number (KHN) of each sample.

### Color stability

Color measurements were carried out under identical environmental conditions throughout all assessments. Each specimen was evaluated using the CIE Lab* system, where L* indicates luminosity (0 = black; 100 = white), a* ranges from red (+a*) to green (-a*), and b* from yellow (+b*) to blue (-b*). Readings were taken against a black background to determine color stability. The overall color difference (ΔE*) was then calculated according to the standard equation ^18^:

$${{\Delta E}^{*}}_{ab}=\;{\left[{\left({\Delta L}^{*}\right)}^{2}+{\left({\Delta a}^{*}\right)}^{2}+{\left({\Delta b}^{*}\right)}^{2}\right]}^{1/2}$$

In this formula, ΔL*, Δa*, and Δb* represent the differences between final and baseline values for the L*, a*, and b* coordinates, respectively. ΔE* was determined both after 24 h and following the aging procedures (mechanical brushing or thermocycling). For each specimen, three readings were taken, and their mean was used as the representative value. The color differences were calculated using the CIE Lab* system (ΔEab) due to its wide use in dental research, ease of calculation, and comparability with previous studies ^19^ ^,^ ^20^ ^,^ ^21^. The ΔE*ab formula is simple and intuitive, facilitating data processing, reproducibility, and interpretation of results in a research context.

### Gloss evaluation

Gloss was assessed with a glossmeter (Novo Curve, Rhopoint Instruments Ltd, Sussex, UK) calibrated at a 60° incidence angle ^22^. The device, with a 4.5 mm aperture, was standardized on black glass supplied by the manufacturer before use. For each specimen, three measurements were taken, rotating the sample 90° between readings; the mean of these values was considered the gloss result. Outcomes were expressed in gloss units (GU).

### Mechanical brushing

Mechanical brushing was carried out using powered toothbrushes (Oral-B Pro-Health Power, Procter & Gamble, Brazil) mounted on a stationary support. The brush head included three types of bristles arranged at different angles and heights. Thirty resin composite discs were brushed for 90 minutes at room temperature with a constant load of 1.96 N. Based on an average of three daily brushings of 5 s per surface, this protocol corresponded to approximately one year of brushing (10,000 cycles per specimen) ^23^. A slurry was prepared by mixing Colgate Total 12 dentifrice (Colgate-Palmolive Company, Brazil) with distilled water in a 1:2 weight ratio (100 g dentifrice + 200 ml water; ISO 14569-1). The solution was freshly prepared each day, 20 minutes before use. During brushing, 1.0 ml of slurry was manually applied every 30 s between the specimen surface and the brush. After the procedure, samples were rinsed under running water.

### Thermocycling

For artificial thermal aging, 10 specimens from each group (control, WR, and MR) were submitted to a thermocycling protocol (MSCT-3, Marcelo Nucci ME, Brazil) comprising 10,000 cycles. Each cycle consisted of immersion in distilled water at 5 °C for 10 s, followed by immersion at 55 °C for 10 s, with a transfer interval of 5 s between baths.

### Final evaluations and statistical analyses

After brushing or thermocycling, all specimens were re-evaluated for microhardness, color stability, and gloss using the same methods previously described. Statistical analyses were conducted with SPSS software (v.17.1; IBM SPSS Statistics for Windows, v.21.0, IBM Corp., Armonk, NY, USA), adopting a 5% significance level. Data distribution and homogeneity were verified with the Shapiro-Wilk test (p > 0.05). Surface microhardness was analyzed using two-way repeated measures ANOVA (split-plot design) followed by Tukey’s post hoc test (α = 0.05). For ΔE and gloss, the Kruskal-Wallis test was applied (α ( 0.001), followed by the Dunn post-hoc test.

## Results

Table 2 presents the Knoop microhardness results after aging (toothbrushing and thermocycling). The original data were subjected to the Shapiro-Wilk normality test, resulting in non-normal values (p < 0.03). Therefore, the nonparametric Kruskal-Wallis ANOVA test and Dunn's post-hoc test were applied for comparisons between groups, and the Wilcoxon test was used for comparisons before and after aging (α = 5%). At the initial time, the groups not covered by modeling liquids obtained higher microhardness when compared to groups with wetting agents, followed by WR groups, while MR groups presented the lowest microhardness values. Following mechanical toothbrushing, the control group exhibited a slight numerical decrease relative to its initial value without statistical significance. However, this group maintained values superior to those of the experimental groups. The MR group showed a statistically significant increase in microhardness after brushing, whereas the WR group presented only a numerical increase without statistical significance. After thermocycling, the control group also showed a slight numerical decrease relative to baseline without statistical significance and remained superior to the experimental groups, which again did not differ significantly from each other. Only the MR group exhibited a statistically significant increase in microhardness following thermocycling.

For the assessment of color alteration (Table 3) after aging (toothbrushing and thermocycling), the ΔE values of the specimens were subjected to the Shapiro-Wilk normality test, which indicated non-normal distributions (p < 0.05). Consequently, the Kruskal-Wallis test was employed for intergroup comparisons (α = 5%). Following mechanical toothbrushing, the control group and the MR experimental group did not exhibit statistically significant changes in ΔE, whereas the WR group presented a statistically significant variation in color. After thermocycling, the Kruskal-Wallis test also revealed a significant effect (p < 0.001), requiring Dunn’s post-hoc test. According to this analysis, the greatest color variation was observed in the MR experimental group.

**Table 2:** Means (± standard deviation) and medians of microhardness (KHN) of resin composite, covered or not by wetting agents, before and after mechanical brushing and thermocycling.

	Groups	Baseline	After aging
Toothbrushing	Control	81.3 (±25.2) 71.2 Aa	73.0 (±23,8) 68.3 Aa
	Wetting Resin (wr)	21.0 (±0.98) 21.0 Bb	27.5 (±5.44) 26.3 Bb
	Modeling Resin (MR)	2.34 (±1.12) 2.15 Cc	22.5 (±10.8) 18.5 Bb
Thermocycling	Control	86.1 (±13.3) 86.8 Aa	69.3 (±4.37) 69.6 Aa
	Wetting Resin (wr)	22.6 (±2.59) 22.8 Bb	22.1 (±2.91) 22.7 Bb
	Modeling Resin (MR)	1.42 (±0.74) 1.05 Cc	20.2 (±8.48) 20.2 Bb

Values followed by distinct letters differ from each other (p≤0.05). Uppercase letters compare microhardness before and after aging, while lowercase letters compare microhardness between groups during the same aging.

**Table 3:** Means (± standard deviation) and medians of ΔE of a nanohybrid resin composite, with or without the application of wetting agents, after brushing cycle and thermocycling.

	Groups	ΔE
Toothbrushing	Control	1.39 (±0.58) 1.28 B
	Wetting Resin (wr)	18.0 (±0.68) 18.1 A
	Modeling Resin (MR)	1.13 (±0.84) 0.82 B
Thermocycling	Control	1.50 (±0.55) 1.40 B
	Wetting Resin (wr)	2.05 (±0.67) 2.20 B
	Modeling Resin (MR)	4.60 (±1.33) 4.27 A

Distinct letters differ from each other at the same aging (p≤0.05).

For the assessment of specimen gloss following mechanical toothbrushing, the Shapiro-Wilk and Levene tests indicated normal and homoscedastic samples for the toothbrushing data (p > 0.05), thus permitting the application of a two-way ANOVA and Tukey’s post hoc test. Table 4 presents the mean and standard deviation values for the initial gloss and the gloss values after each aging method. At the initial gloss measurement, the specimens from the WR and MR groups exhibited higher gloss values than the control group. After mechanical toothbrushing, the control group presented the lowest gloss values when compared to WR and MR. In addition, only the MR group showed a statistically significant increase in gloss after brushing when compared to its baseline value. For the thermocycling data, the Shapiro-Wilk test indicated a non-normal distribution (p = 0.03), allowing the use of the nonparametric Kruskal-Wallis test with Dunn’s post hoc test for intergroup comparisons and the Wilcoxon test for intragroup comparisons. Following thermocycling, both experimental groups demonstrated significantly lower gloss values compared to baseline, while the control group maintained similar gloss before and after aging.

**Table 4:** Means (± standard deviation) and medians (to thermocycling) of surface gloss (GU) of a nanohybrid resin composite, with or without the application of wetting agents, after brushing cycle and thermocycling.

	Groups	Baseline	After aging
Toothbrushing	Control	11.8 (±1.62) Ab	12.9 (±3.94) Ab
	Wetting Resin (wr)	17.2 (±8.26) Aa	23.2 (±7.68) Aa
	Modeling Resin (MR)	17.3 (±3.09) Ba	25.7 (±5.50) Aa
Thermocycling	Control	11.7 (±1.23) 11.55 Aa	11.5 (±0.97) 11.7Aa
	Wetting Resin (wr)	15.2 (±5.71) 16.05 Aa	7.31 (±1.07) 7.50 Bb
	Modeling Resin (MR)	14.6 (±6.21) 12.7 Aa	8.20 (±1.88) 8.35 Bb

Values followed by distinct letters differ from each other (p≤0.05). Uppercase letters compare gloss before and after aging, while lowercase letters compare gloss between groups.

## Discussion

At baseline, specimens coated with a wetting agent showed lower Knoop microhardness values than those without modeling liquids. Commercial modeling liquids are typically comprised of high amounts of TEG-DMA, which is a recognized monomer with diluting ability, probably due to its low viscosity, hydrophilic behavior, and high reactivity characteristics, so the application of TEG-DMA-based commercial liquids could cause a softening effect on the organic matrix of restoration, perhaps explaining this trend towards reduced hardness as compared to the non-modeled resin composite ^24^.

Among the groups, MR exhibited the lowest medians. After brushing and thermocycling, however, the WR group maintained its hardness values and no longer differed from MR. Therefore, the first null hypothesis was rejected.

The absence or low percentage of filler content in wetting agents can explain the lower microhardness when compared to control groups not covered by modeling liquids. Furthermore, MR is a medium-viscosity resin composite composed of UDMA, Bis-EMA, TEGDMA, and Bis-GMA, whereas WR contains equal amounts of Bis-GMA and TEGDMA, providing superior mechanical behavior compared with conventional dental adhesives ^25^. The TEGDMA monomer makes an efficient hydrogen bond acceptor for the Bis-GMA monomer; therefore, multiple hydrogen bonds that stabilize each other allow more cohesive and densely packed performance to the WR group, resulting in higher surface hardness values, while MR composition may alter the physicochemical stability of the material surface due to the possibility of residual monomer release, which impairs mechanical properties and facilitates crack propagation within the material (10, 24, 26). The controversial microhardness increase of MR after aging can be explained by the effect of toothbrushing abrasion and partial dilution of MR ingredients over time, resulting in a thinner layer of modeling material. Furthermore, some areas of the surface of the nanohybrid resin composite may have been exposed, which has better mechanical properties. The filling particles of silicon and zirconia dioxide presented in the nanohybrid resin composition may have resulted in an increase in the average of superficial microhardness, since filler particles have natural properties that increase the hardness of resin composites via intense ionic interatomic bonds ^27^. However, the exposed surface after aging likely consisted of a heterogeneous interface between residual modeling resin and partially uncovered composite matrix, rather than a fully exposed composite surface. Therefore, the mechanical performance would not necessarily reach values comparable to the control group, which did not receive a modeling resin layer.

Further complementary analyses, such as the evaluation of surface roughness, sorption, and solubility of the modeling liquids, would contribute to elucidating this hypothesis, since it would be possible to analyze the surface layer and the possibility of accelerated hydrolytic degradation and leaching of monomers.

Bayraktar et al. ^13^ ^)^ also reported a decrease in the Vickers microhardness value (VHN) of six resin-based composites after modeling resin application prior to light curing. The wetting agents might have produced a high resin-rich layer on the surface of the nanohybrid resin composite. Consequently, the filler content of the final composite layer might be directly related to the VHN values ^28^. Additionally, Tuncer et al. ^14^ reported that the application of Modeling Resin's application (Bisco, USA) was able to reduce the hardness of nanohybrid resin composite (GrandioSO, VOCO, Germany) and a microhybrid resin composite (Gradia Direct Posterior, GC America, USA). Although the absolute values of Knoop microhardness are slightly different from those of Vickers microhardness, the relative hardness trend among different materials remains consistent, allowing for reliable comparison between groups. It has already been demonstrated that values obtained using the Knoop hardness test showed a high correlation with Vickers hardness measurements ^29^.

Regarding color stability, our results were aging and wetting agent-dependent. Therefore, the second null hypothesis must be rejected. After the brushing cycle, the control and MR groups did not show significant ΔE, whereas the WR group presented a very high ΔE. These results are consistent with those reported by Pereira et al. ^30^ when MR was submitted to 10,000 brushing cycles and showed no statistical difference in color change. The high ΔE achieved by WR disagreed with the findings of a meta-analysis review ^31^ of modeler liquids on the properties of direct resin-based composites that concluded that modeled and non-modeled resin composites showed similar results in terms of color change. Regarding WR specifically, Maalekipour et al. ^32^ observed that WR released high amounts of TEGDMA and UDMA in water and methanol medium, respectively, after 24 hours. This elution of components from the resin composite mass may have affected the ΔE of the nanohybrid resin composite. After thermocycling, MR presented the greatest color variation. Tuncer et al. ^14^ concluded that the Bisco Modeling Resin (Bisco, Schaumburg, IL, USA) did not affect the color of six different resin composites after 10,000 thermocycles. To date, no studies have directly evaluated WR or MR agents after thermocycling for direct comparison with our results. 

Regarding another optical property, the modeling liquids initially fulfilled their desirable function of enhancing the resin composite gloss. Overall, the application of wetting agents increased the initial gloss values compared to the control group. One possible explanation lies in the fact that modeling liquids could have a slight diluting effect on the organic matrix of the uncured resin composite, decreasing its viscosity during anatomical sculpture; therefore, the better fluidity of the resin composite reduced gap formation, ultimately resulting in a more homogeneous and glossier surface ^24^. However, following thermocycling, these values decreased significantly in the experimental groups and remained stable in the control group (without wetting agents). This indicates that the modeling liquids probably do not present the ability to maintain the gloss of restorations over time under oral cavity conditions. Nevertheless, after mechanical brushing, the observed outcome was reversed. Specifically, the experimental groups exhibited higher gloss values when compared to the initial results and the control group, thus demonstrating a positive result. A possible explanation for these findings may be related to the mechanical brushing process, contributing to a greater polishing of the restoration. In line with our results, Pereira et al. ^30^ demonstrated that the application of a modeling resin helped preserve surface gloss of resin composite restorations after brushing and staining challenges, reinforcing the notion that such agents may offer short-term aesthetic benefits by smoothing the surface and filling in superficial irregularities. Thus, the third null hypothesis must be rejected. 

It should be noted that in many study methodologies, the process included polishing the material's surface using sandpaper ^13^ ^,^ ^30^. This procedure may have resulted in the removal of the most superficial layer with modeling resins, leading to the partial or total exposure of the untreated subsurface resin composite. This study aimed to analyze the real effects of the material, given that there is no clinical standardization of the application of wetting agents, and it is difficult to control the amount of liquid and the thickness of the layer. 

Some limitations should be acknowledged. As with in vitro studies, the artificial nature of laboratory simulations may not fully reproduce the complex conditions found in the oral environment. The study evaluated a single nanohybrid resin composite and two specific wetting agents, which may limit the generalizability of the findings to other materials. Moreover, while microhardness, color stability, and gloss were analyzed, complementary assessments—such as surface roughness, water sorption, or topographic imaging—were not included and could provide further insights into material performance. Nonetheless, the protocols adopted allowed for consistent comparison among groups, and the findings contribute relevant data regarding the behavior of surface sealants under simulated aging conditions. It is important to conduct more detailed studies on these materials since modeling resins can impact the durability, aesthetics, and functional performance of resin composite restorations.

Wetting agents’ application presented a deleterious influence on Knoop superficial microhardness and on color stability, although results were material and aging-dependent. When covered by modeling liquids, the resin composite gloss increased after brushing but decreased after thermocycling, while the resin composite with no wetting agents’ application remained with stable gloss. The results of the present study discourage the application of wetting agents in nanohybrid composites.

## Data Availability

The research data are available upon request.
